# Sirtinol regulates the balance of Th17/Treg to prevent allograft rejection

**DOI:** 10.1186/s13578-017-0182-2

**Published:** 2017-10-27

**Authors:** Qing Ye, Mingjian Zhang, Yang Wang, Shangxi Fu, Shu Han, Liming Wang, Quanxing Wang

**Affiliations:** 1 Institute of Organ Transplantation, Changzheng Hospital, Second Military Medical University, Shanghai, China; 2 National Key Laboratory of Medical Immunology, Second Military Medical University, Shanghai, China; 3 Department of Clinical Surgery, Chinese PLA General Hospital, 28th Fuxing Road, Beijing, China

**Keywords:** Sirtinol, Allograft rejection, Th17/Treg

## Abstract

**Background:**

Current immunosuppressive medications used after transplantation induce significant toxicity , and a new medication regimen is needed. Based on recent research, Sirt1 exerts a proinflammatory effect on the immune response. Sirtinol is a Sirt1 inhibitor, but its impact on allograft rejection and its molecular mechanisms of action have not yet been reported.

**Resluts:**

In this study, we examined the effect of sirtinol on prolonging allograft survival in a mouse cervical heterotopic heart transplantation model. Based on an examination of the allograft, allografts from sirtinol-treated recipients show significantly lower levels of IL-17A expression and higher levels of Foxp3 expression. In vivo, sirtinol reduces the proportion of Th17 cells and increases the proportion of Treg cells in splenocytes from recipients. In vitro, sirtinol reduces the proportion of Th17 cells and decreases the expression of IL-17A and RORγt in an isolated CD4^+^ T cell population. Moreover, we identified synergistic effects of sirtinol and FK506 on prolonging allograft survival, and sirtinol synergizes with FK506 to promote Foxp3 expression.

**Conclusion:**

Sirtinol, a Sirt1 inhibitor, may be a promising immunosuppressive drug to prevent the rejection reaction in combination with FK506.

## Background

Organ transplantation is the final therapeutic schedule for most patients with end-stage organ disease. According to numerous studies, calcineurin inhibitors (CNIs) significantly improve short-term solid organ graft survival in transplant recipients. The rate of acute rejection has been dramatically reduced and the 1-year survival rate has clearly increased since CNIs have been administered to patients after transplantation. However, the long-term used of CNIs might result in various side-effects, such as renal toxicity, neurotoxicity and increased risks of infection and cancer [1]. CNIs are now thought to be necessary for preventing allograft rejection, but reduced doses are preferred [2].

Based on traditional transplantation immunology, APCs from donors or recipients activate T cells through direct recognition and indirect recognition, respectively. Next, T helper cells release interferon (IFN)-γ, interleukin (IL)-2, tumor necrosis factor (TNF) and other types of cytokines to mediate rejection [3, 4]. Therefore, CNIs are administered with FKBP12 to inhibit the activation of nuclear factor of activated T-cells (NF-AT) and the subsequent transcription of the IL-2 gene to prevent the reject reaction.

The Th17/Treg balance was strongly associated with allograft rejection in some recent studies [5, 6]. Th17 cells are important for mediating acute and chronic rejection, whereas Tregs contribute to the induction and maintenance of tolerance of the allografts in recipients [7, 8]. However, CNIs do not effectively restrain the rejection reaction mediated by Th17 cells, and CNIs have been reported to cause a Th17/Treg imbalance [9]. Therefore, therapeutic strategies aimed at manipulating the Th17/Treg balance are now considered the most promising approach for preventing allograft rejection.

In recent years, immunologists have paid more attention to epigenetic regulation, which is thought to be the precise regulatory mechanism in immune cells. Transplant scientists focus on the mechanism by which epigenetics regulate rejection, particularly by preventing chronic rejection. Sirtuins are NAD-dependent class III histone deacetylases (HDACs) that play critical roles in diverse physiological processes, such as prolonging the life-span, metabolism, cell senescence, cell autophagy/apoptosis, autoimmunity, oxidative stress, and inflammation [10]. Sirtuins include seven members known as Sirtuin1-Sirtuin7. Among them, Sirtuin1 (Sirt1) specifically acts as an epigenetic regulator that modulates the activity of several transcription factors important for immune function.

Sirt1 was initially suggested to exert a primarily anti-inflammatory function [11–13]. However, recent research focusing on T cells has shown that Sirt1 exerts a proinflammatory effect on the immune response. On one hand, Sirt1 was identified as a negative regulator of Treg cell function by deacetylating Foxp3, the signature transcription factor of Treg cells [14, 15]. On the other hand, Sirt1 might positively regulate the function of Th17 cells by modulating RORγt activity [16]. Based on the findings from these studies, Sirt1 may be a new potential therapeutic target to promote immunosuppression in transplant recipients.

As a Sirt1 inhibitor, sirtinol has displayed anti-tumor [17–19] and anti-inflammatory properties [20, 21], but its impact on allograft rejection and its molecular mechanisms of action have not yet been reported. In the present study, we established a mouse model of cervical heterotopic heart transplantation and evaluated the impact of sirtinol on cardiac allograft rejection. Notably, sirtinol synergizes with tacrolimus to prevent mouse cardiac allograft rejection, as sirtinol inhibits the Th17-mediated rejection and increases the proportion of Treg cells.

## Methods

### Mice and reagents

Mice were obtained from the Second Military Medical University Animal Supply Center and housed in a specific pathogen-free facility. C57BL/6 (aged 10–14 weeks, male) mice were used as recipients and BALB/C mice (aged 8–12 weeks, male) were used as heart donors. All animal experiments were conducted under the experimental protocol approved by the Ethics Review Committee for Animal Experimentation of the Second Military Medical University. We purchased sirtinol and tacrolimus from Selleck Chemicals (Boston, MA, USA). Sirtinol and tacrolimus were dissolved in dimethyl sulfoxide (DMSO), and DMSO was used as a control.

### Heart transplantation model

The cervical heterotopic heart transplantation mouse model was prepared as previously described [22]. Recipient mice were intraperitoneally injected with sirtinol (1 mg/kg/day), FK506 (1 mg/kg/day), or both sirtinol (1 mg/kg/day) and FK506 (1 mg/kg/day). The study point being evaluated was defined as complete cessation of the heartbeat. Survival rates of cardiac grafts were monitored by palpation.

### Histology examination

Cardiac allografts were excised at 7 or 12 days post-transplantation, and fixed with 5% neutral paraformaldehyde, routinely processed, and embedded in paraffin. Histological sections for light microscopy were cut to a thickness of 4 μm and stained with hematoxylin and eosin. Infiltrating cells were counted under at high magnification (× 40) using ImagePro Plus software.

### RNA isolation, reverse transcription, and quantitative PCR

Total RNA was extracted from allografts using TRIzol reagent (Invitrogen, Carlsbad, CA, USA), according to the manufacturer’s instructions; cDNAs were synthesized using an oligo d(T) primer (Applied Biosystems, Foster City, California, U.S.) and a Superscript III Reverse Transcriptase Kit (Invitrogen). A StepOne™ Real-Time PCR System (Applied Biosystems) and a SYBR RT-PCR kit (Takara, Japan) were used for the quantitative real-time PCR analysis. All reactions were conducted in a 20 μl reaction volume in triplicate. The relative expression levels of a target gene were normalized to GAPDH expression. The specificity of qPCR was verified with a melting curve analysis and agarose gel electrophoresis. Primer sequences used in the RT-PCR analysis are shown in Table 1 [23].

**Table 1:** RT-PCR primer sequence

Primer	Sequence
RORγt	Forward 5′-TCA CCT GAC CTA CCC GAG G-3′
	Reverse 5′-TCC AAG AGT AAG TTG GCC GTC-3′
GAPDH	Forward 5′-AGG TCG GTG TGA ACG GAT TTG-3′
	Reverse 5′-TGT AGA CCA TGT AGT TGA GGT CA-3′
IL-17A	Forward 5′-TCG CCA TTC AGC AAG AAA TCC-3′
	Reverse 5′-CAC AGG TGC AGC CAA CTT TTA-3′
IFN-γ	Forward 5′-GAA CTG GCA AAA GGA TGG TGA-3′
	Reverse 5′-TGT GGG TTG TTG ACC TCA AAC-3′
IL-4	Forward 5′-GGT CTC AAC CCC CAG CTA GT-3′
	Reverse 5′-GCC GAT GAT CTC TCT CAA GTG AT-3′
FOXP3	Forward 5′-TCA AGT ACC ACA ATA TGC GAC C-3′
	Reverse 5′-CCA TCG GAT AAG GGT GGC A-3′

### T cells isolation and flow cytometry

We isolated T lymphocytes from spleens. After preparing a single cell suspension and counting the cells, we used magnetic beads (Miltenyi Biotec) to isolate CD4^+^ cells. Isolation was performed in the dark, and the total number of cells added to the LS column was restricted to 1 × 10^8^. Anti-CD4, anti-CD25, anti-IFN-γ, anti-IL-4, anti-IL-17A, and anti-Foxp3 antibodies were purchased from BioLegend. Intracellular straining for IFN-γ, IL-4, IL-17A and Foxp3 was performed using the Prem/Fix Buffer set, according to the manufacturer’s instructions [24]. After staining, cells were suspended in PBS + 1% FBS and analyzed using an Accuri C6 flow cytometer from BD Biosciences (San Jose, CA, USA). We also detected the serum IFN-γ, IL-4, and IL-17 levels using enzyme-linked immunosorbent assay (ELISA) kits, according to the manufacturer’s protocol (R&D Systems, Minneapolis, MN, USA).

### Statistical analysis

Data were analyzed with GraphPad Prism 5.0 software (GraphPad, Inc., La Jolla, CA, USA). All normally distributed data were displayed as means ± standard deviations (SD). Measurements between two groups were analyzed using Student’s *t* test or the Mann–Whitney U test. Allograft survival was assessed using a log-rank (Mantel-Cox) test. A P value < 0.05 was considered significant.

## Results

### Sirtinol prolongs the survival of cardiac allografts

**Fig. 1:**
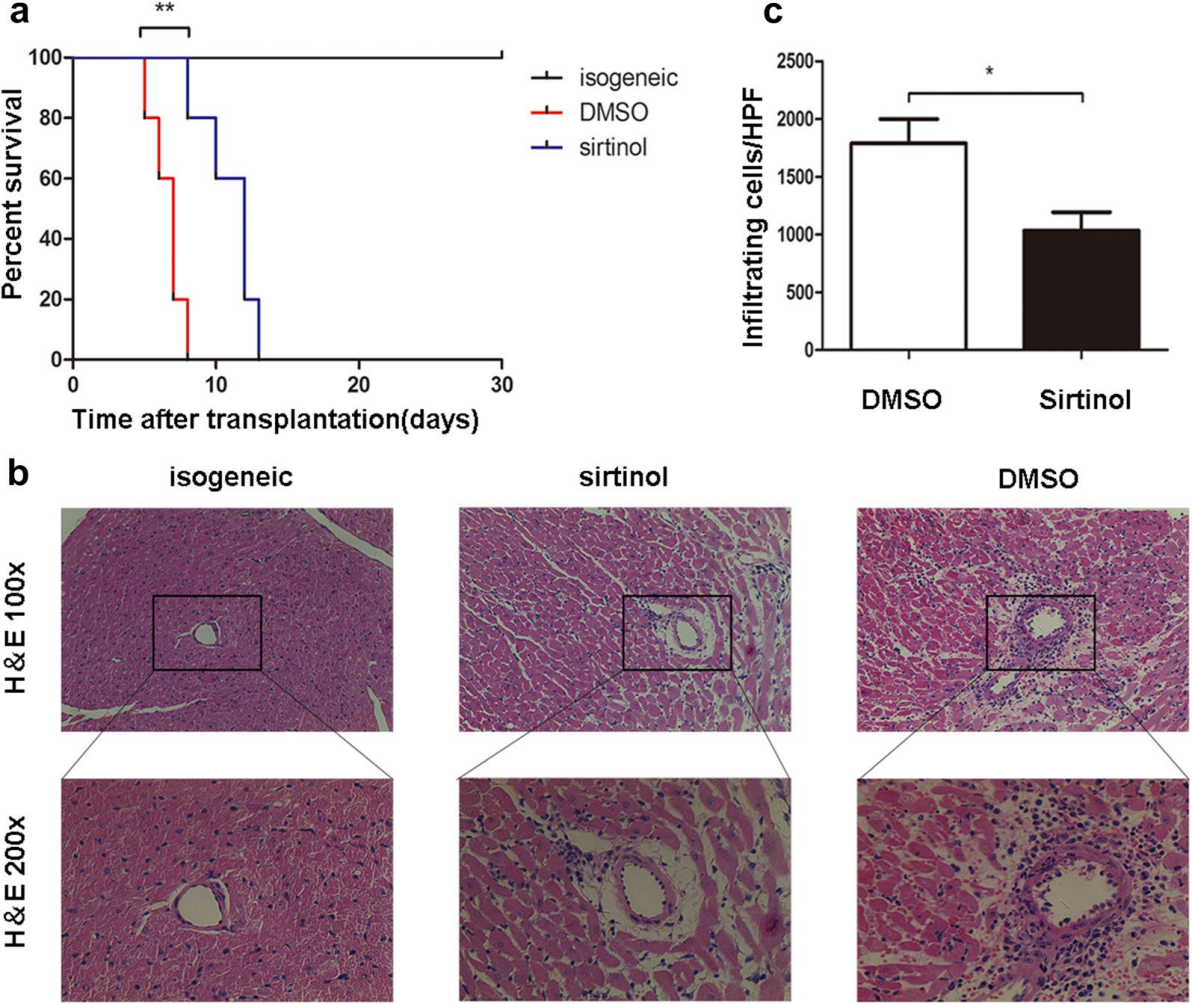
Sirtinol prolongs the cardiac allograft survival in murine cervical heterotopic cardiac transplant model. **a** Fully MHC-mismatched cardiac allograft recipients (BALB/C to C57BL/6) were treated with DMSO, sirtinol (1 mg/kg/day) by intraperitoneal injection since the day receiving transplantation until when cardiac arrest occurred. Graft survival was assessed every day. **b** Pathological examination of allografts harvested on day 7 post-transplantation. Shown are section graphs from the low power field and high power field. **c** Infiltrating cells per high field were counted from five mice per group. *P < 0.05, **P < 0.01, compared with DMSO group

We developed a fully MHC-mismatched murine cervical heterotopic cardiac transplant model in which C57BL/6 mice were transplanted with the hearts from BALB/C mice and randomized to treatment with sirtinol (1 mg/kg/day i.p. after transplantation) or DMSO as a vehicle control to assess the effect of sirtinol on immune responses. The median survival time of the DMSO group was 7 days, but the administration of sirtinol prolonged the median survival of the allograft to 10 days (Fig. 1a).

Next, five allografts from each experimental group were harvested on day 7 post-transplantation and subjected to histological analysis. The histological analysis clearly revealed a rejection reaction in the DMSO group, whereas the sirtinol group was characterized by less infiltration (Fig. 1b, c).

Based on these results, sirtinol prolongs the survival of MHC fully mismatched cardiac allografts, but the exact mechanism remains unknown.

### Sirtinol regulates the Th17/Treg balance

**Fig. 2:**
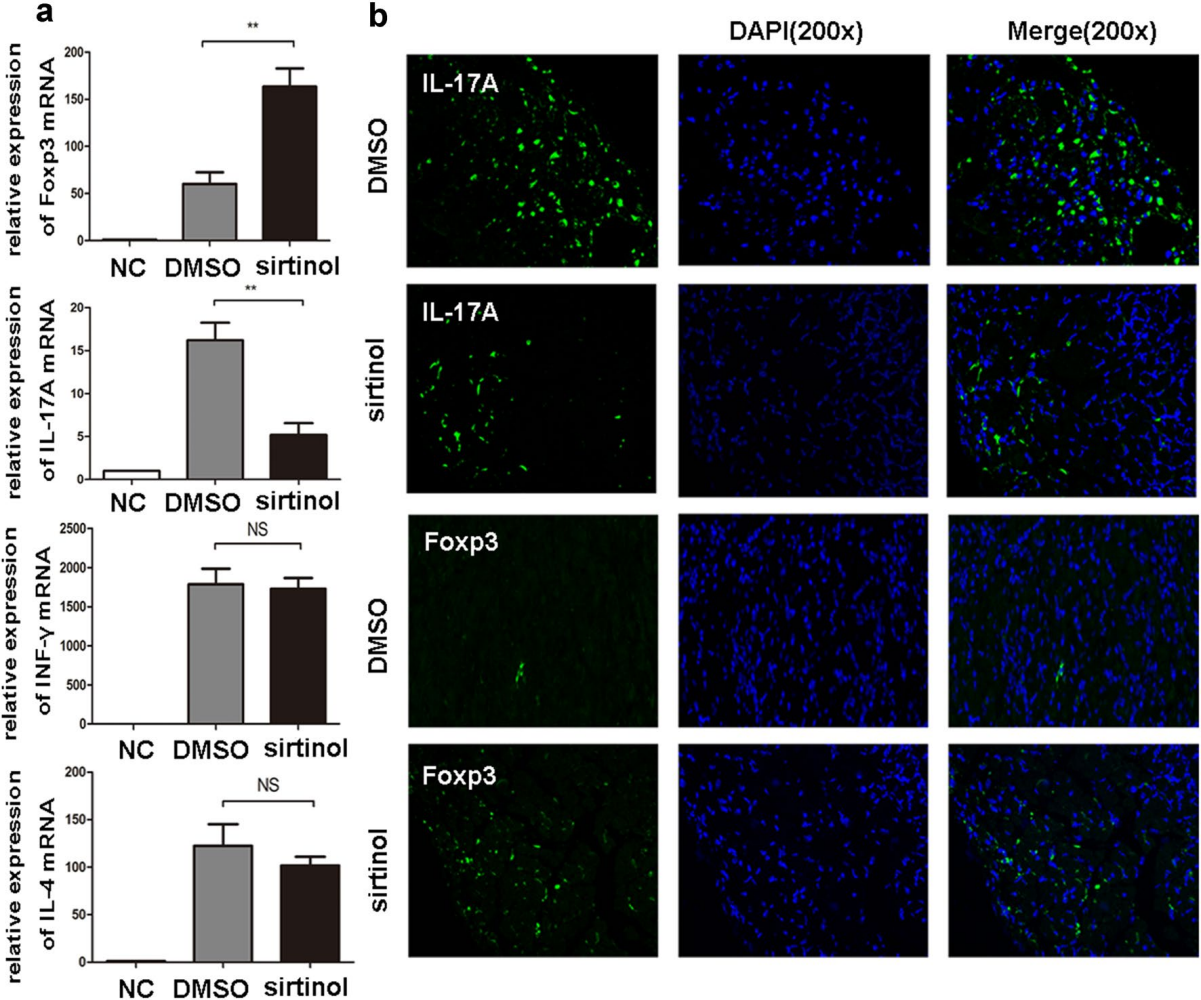
The effect of sirtinol on the expressions of inflammatory cytokines. **a** The allografts were harvested 7 days after transplantation. IL-17A, Foxp3, IFN-γand IL-4 mRNA were measured by qPCR. The data are expressed as mean ± SD (n = 5). *P < 0.05, **P < 0.01, compared with DMSO group. NC, negative control represented cardiac graft from isotransplantation. **b** Immunofluorescence examination of allografts harvested on day 7 post-transplantation. Shown are section graphs from high power field

Next, we examined the impact of sirtinol on the expression of inflam-matory cytokines and regulatory molecules. Allografts from the DMSO and sirtinol groups were harvested on day 7 after transplantation for quantitative PCR analysis. T cells play a primary role in the acute rejection reaction, and thus we first evaluated the expression of IFN-γ, IL-4, IL-17A and Foxp3. Compared with allografts derived from the DMSO group, allografts from sirtinol-treated recipients showed significantly lower levels of IL-17A and higher Foxp3 expression. In contrast, the expression of IFN-γ and IL-4 was not significantly different between the DMSO group and sirtinol group (Fig. 2a). The immunofluorescence analysis also revealed the same results (Fig. 2b).

**Fig. 3:**
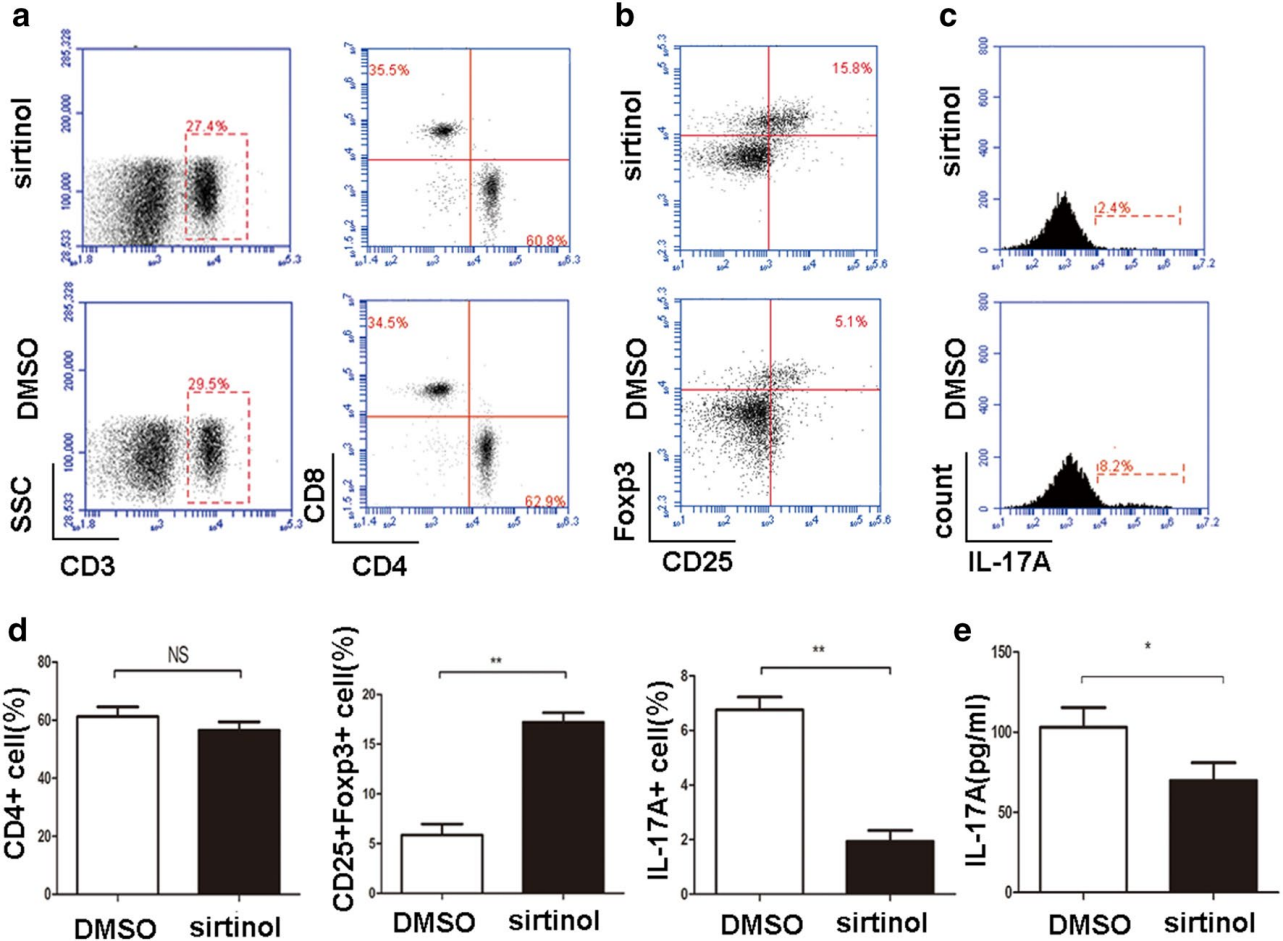
The effect of sirtinol on the proliferation and differentiation of T cells subset. **a** The recipient spleens were harvested 7 days after transplantation. CD3^+^ T cells, CD4^+^ T cells and CD8^+^ T cells from splenocyte were measured by flow cytometry. **b** The recipient spleens were harvested 7 days after transplantation. CD4^+^ T cells were isolated and CD25^+^ and Foxp3^+^ T cells were measured by flow cytometry. Shown are representative of five separate experiments. **c** The recipient spleens were harvested 7 days after transplantation. CD4^+^ T cells were isolated and IL-17A^+^ T cells were measured by flow cytometry. Shown are representative of five experiments. **d** Percentages of CD4^+^, CD25^+^Foxp3^+^ and IL-17A^+^ cells were presented. **e** ELISA assays were performed to test the IL-17A levels on day 7 after transplantation. The data are expressed as mean ± SD (n = 5). *P < 0.05, **P < 0.01, compared with DMSO group

Given the role of Foxp3 in Treg cells, we evaluated the number of Treg cells in hosts from the DMSO and sirtinol groups. We isolated cells from spleen at 7 days after transplantation and then analyzed cells using flow cytometry. Indeed, the sirtinol treatment significantly increased the proportion of Treg cells among CD4^+^ T cells in the spleen (Fig. 3b). The effects of sirtinol on cytokine production, including IL-4, IFN-γ and IL-17A, were also assessed using intracellular staining. A significant decrease in IL-17A staining was observed in the sirtinol group (Fig. 3b). No obvious changes in IL-4 and IFN-γ expression were observed between the DMSO group and sirtinol group. We also detected the serum IFN-γ, IL-4, and IL-17A levels using ELISA kits and obtained the same results (Fig. 3c).

**Fig. 4:**
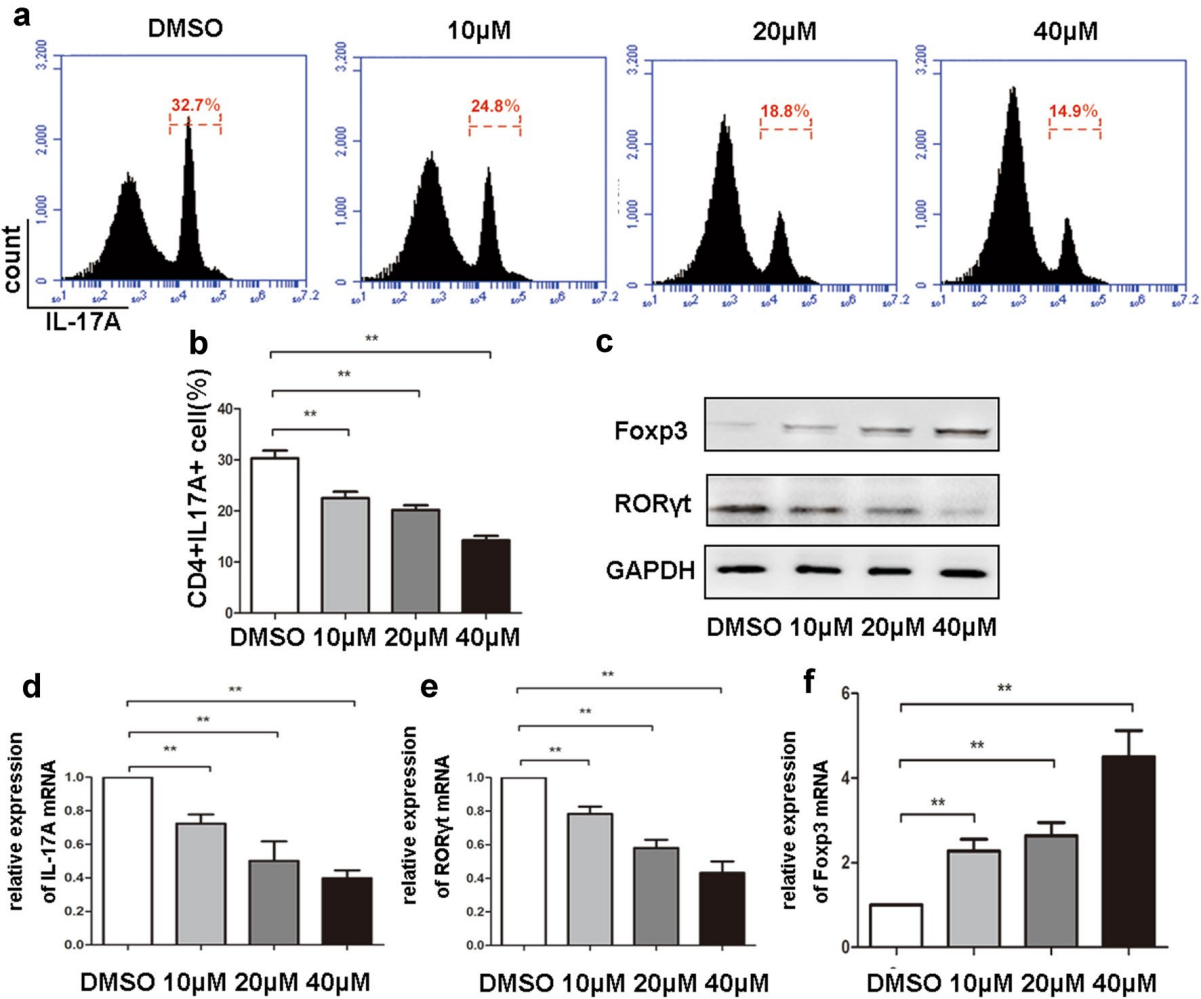
Sirtinol regulates Th17/Treg balance. **a** Naive CD4^+^ T cells were isolated from mouse spleen, and polarized for Th17 differentiation in the presence different concentration of sirtinol. IL-17A expressing CD4^+^ T cells were measured by flow cytometry. Shown are representative of five separate experiments. **b** Percentages of IL-17A^+^ cells were presented. **c** The effect of sirtinol on the expressions of RORγt and Foxp3 measured by western blots. Shown are representative of five separate experiments. **d**–**f** Gene expressions of IL-17A, RORγt, Foxp3 in CD4^+^ T cells measured by qPCR. The data are expressed as mean ± SD (n = 5). *P < 0.05, **P < 0.01, compared with DMSO group

Sirtinol suppressed IL-17 expression in allografts; thus, we examined the impact of sirtinol on the differentiation of Th17 cells. Naive CD4^+^ T cells were isolated and then polarized to the Th17 lineage in the presence of sirtinol or DMSO, as previously described [25]. According to the results of the flow cytometry analysis of polarized T cells, sirtinol significantly suppressed the production of Th17 cells (Fig. 4a), and the western blot analysis indicated that sirtinol reduced the expression of RORγt and promoted Foxp3 expression (Fig. 4b). The qPCR analysis further confirmed that sirtinol down-regulated IL-17A and RORγt expression, but up-regulated Foxp3 expression (Fig. 4c). Based on these results, sirtinol may regulate the balance between Th17 and Treg cells to prolong cardiac allograft survival.

### Sirtinol functions synergistically with CNIs to alleviate cardiac allograft rejection

**Fig. 5:**
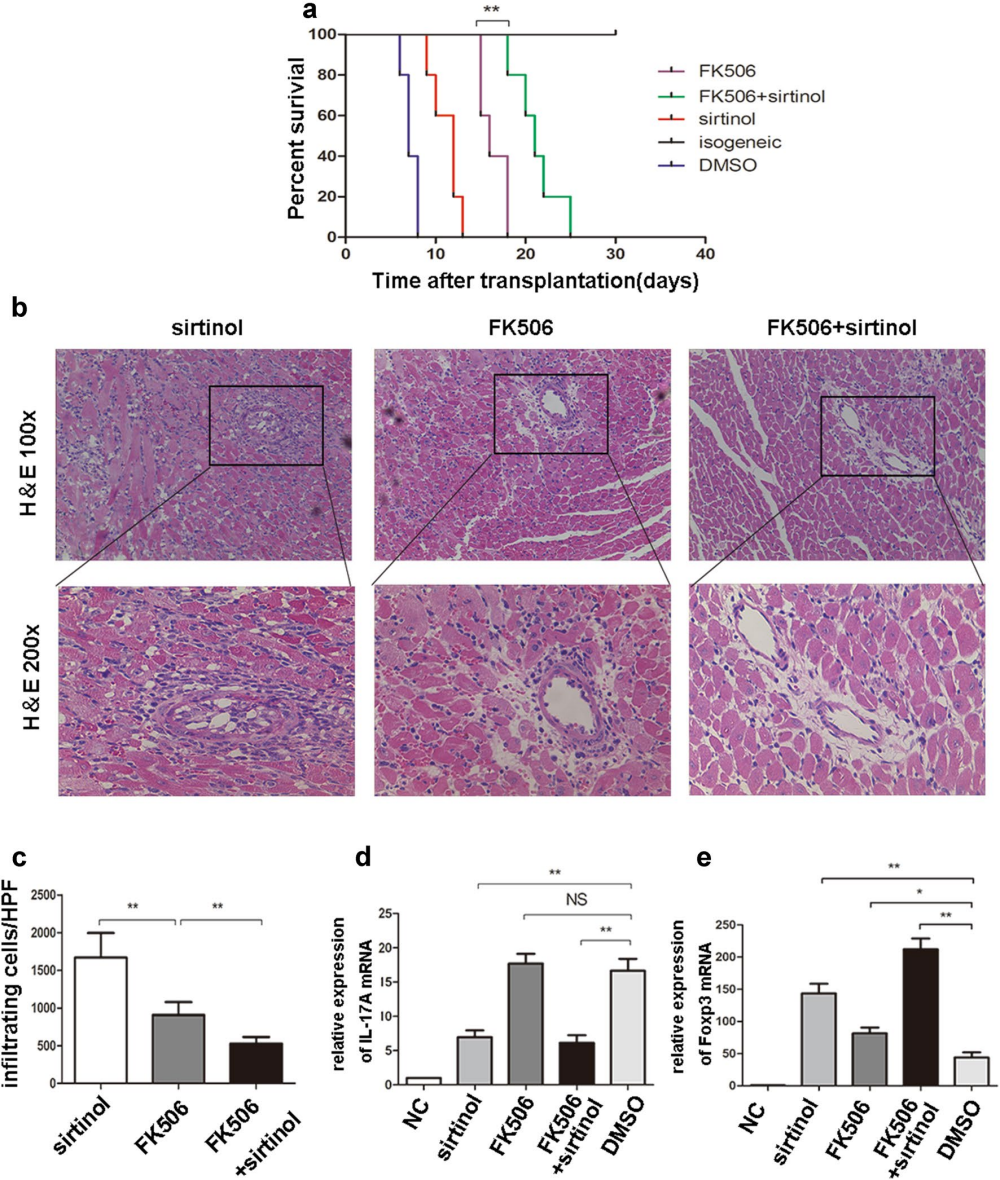
Sirtinol synergizes with FK506 to prevent mouse cardiac allograft rejection. **a** Fully MHC-mismatched cardiac allograft recipients (BALB/C to C57BL/6) were treated with DMSO, sirtinol (1 mg/kg/day), FK506 (1 mg/kg/day) or sirtinol (1 mg/kg/day)—FK506 (1 mg/kg/day) combination by intraperitoneal injection since the day after transplantation until when cardiac arrest occurred. Graft survival was assessed every day. **b** Pathological examination of allografts harvested on day 12 post-transplantation. Shown are section graphs from the low power field and high power field. **c** Infiltrating cells per high power field were counted from five mice per group. **d**, **e** IL-17A and Foxp3 mRNA were measured by qPCR. The data are expressed as mean ± SD (n = 5). *P < 0.05, **P < 0.01. *NC* negative control represented cardiac graft from isotransplantation

Since sirtinol and CNIs use different mechanisms to restrain rejection, we treated mice with sirtinol and tacrolimus after transplantation. Co-administration of sirtinol and tacrolimus prolonged the median allograft survival to 21 days, whereas the administration of tacrolimus prolonged the median allograft survival to 16 days (Fig. 5a).

Next, five allografts from each group were harvested on day 12 post-transplantation and subjected to a histological analysis. Compared with that in the tacrolimus group, co-administration of sirtinol and tacrolimus completely prevented inflammatory infiltration, but did not change myocardial structure (Fig. 5b, c).

We conducted a qPCR analysis to analyze the grafts and confirm these results. Compared with allografts derived from the tacrolimus-treated groups, allografts from the tacrolimus/sirtinol group showed significantly lower levels of IL-17 expression. No significant difference in IL-17 expression was observed between the sirtinol and tacrolimus/sirtinol groups, indicating that sirtinol alone reduced IL-17 expression (Fig. 5d). Interestingly, a synergistic effect of sirtinol and FK506 on Foxp3 expression was detected (Fig. 5e). Based on these data, sirtinol functions synergistically with tacrolimus to promote the survival of cardiac allografts.

## Discussion

Lifelong immunosuppression can result in severe side effects, such as cancer, infection, diabetes, cardiovascular diseases, etc. Currently used immunosuppressive medications cause organ toxicity and do not effectively prevent chronic rejection, limiting long-term allograft survival [26]. Therefore, a new therapeutic target with minimal side effects and adequate immunosuppression is required. In this study, the class III HDAC Sirt1 played a critical role in the T-cell-mediated immune response after organ transplantation, and the inhibition of Sirt1 by sirtinol protected the allograft from inflammatory cell infiltration and prolonged graft survival.

The immunosuppressive effects of conventional Zn-dependent class I and II HDAC inhibitors are widely accepted. The class III HDACs were discovered later than the other two classes, and the role of class III HDACs in immune responses remains controversial. In this study, the Sirt1 inhibitor sirtinol clearly prolonged cardiac allograft survival. Based on the qPCR and flow cytometry results, sirtinol may affect Treg and Th17 cells to limit immune responses.

According to an increasing number of recent studies, Th17 cells play a key role in T-cell-mediated acute rejection. An increased level of IL-17A was observed in the immune rejection reactions in several organs, including the lung and kidney. IL-17A inhibition clearly prolongs allograft survival [27]. In addition, Th17 cells may also participate in chronic rejection. As shown in the study by Yuan et al. Th17 cells mediate chronic inflammation and vasculopathy in the model of cardiac allograft vasculopathy in T-bet^−/−^ recipients [28]. In animals, Th17 cells promote allograft fibrosis by inducing chronic inflammation [29, 30]. Th17 cells secrete IL-17A and IL-21 to mediate chronic rejection [31].

Treg cells, an important T cell subset, also regulate acute rejection post-transplantation. Studies have shown a positive correlation between the population of Treg cells and allograft survival in tissues such as the lung, kidney and liver [32]. In hematopoietic stem cell transplantation, acute or chronic graft-versus-host disease (GVHD) occurs in the mesentery because of low Treg cell counts [33]. Based on research presented above, we consider that the Sirt1 inhibitor sirtinol, which regulates the Th17/Treg balance, has outstanding potential as an immunosuppressive medicine.

Among the immunosuppressive medicines, CNIs are the cornerstone of immunosuppressive therapy post-transplantation and are the main cause of side effects. They inhibit IL-2 synthesis to prevent the activation and proliferation of T lymphocytes. However, the effect of inhibiting Th17 cells is not achieved because of the different activation mechanism of Th17 cells. Thus, we proposed the hypothesis that clinicians should choose a mild immunosuppressant to inhibit Th17 cell-mediated rejection under conditions of long-term CNI use to prolong graft survival. Finally, our research results verify this hypothesis, as the combination of sirtinol and FK506 is more effective at preventing allograft rejection than either of drug alone. Thus, the administration of sirtinol along with a low dose of FK506 may achieve a satisfying immunosuppressive effect with fewer side effects.

FK506, a second generation CNI, inhibits calcineurin activity by a unique, small molecule-mediated, protein–protein interaction. FK506 binds to FKBP12, and this binary complex binds to calcineurin and inhibits its phosphatase activity and downstream signaling. Then, the IL-2 synthesis is inhibited and Th1 cells are not activated because of the lack of IL-2. According to the results from our present study, sirtinol suppresses the production of Th17 cells; therefore, the combination of FK506 and sirtinol should suppress Th cell production more comprehensively than either drug alone. This hypothesis is consistent with our observations in the current study. In addition, CsA and FK506 use different intracellular receptors, but they inhibit cell signaling through similar mechanisms. Calcineurin is a common target of cyclophilin–cyclosporin A and FKBP-FK506 complexes. Thus, we postulate that sirtinol also synergizes with CsA. Our future experiments will verify this hypothesis.

In addition to its immunosuppressive effect, sirtinol also exerts other pharmacological functions. On one hand, sirtinol has been reported to suppress human cytomegalovirus (hCMV) infection and hCMV-induced activation of the molecular mechanisms of senescence and reactive oxygen species (ROS) production; [34] hCMV also causes serious infection in transplant recipients with reduced immune function [35]. On the other hand, pharmacological Sirt1 inhibition by sirtinol exerts a broad anti-tumor effect on several cancers, such as melanoma, breast cancer, and prostate cancer [19, 36–38]. Thus, sirtinol is not only an immunosuppressive agent that can be used to treat allograft rejection, but also a potential drug for preventing hCMV infections and the development of malignant tumors after organ transplantation. However, the side effects of sirtinol must also be considered. In our study, a high-dose sirtinol treatment did not prolong allograft survival and showed unexpected side effects. Therefore, additional studies are needed to evaluate the effects of sirtinol before clinical application.

## Conclusions

Sirtinol prolongs allograft survival by regulating the Th17/Treg balance. Moreover, we identified synergistic effects of sirtinol and FK506 in prolonging allograft survival, and sirtinol synergizes with FK506 to promote Foxp3 expression. Therefore, sirtinol, a Sirt1 inhibitor, may be a promising immunosuppressive drug to prevent rejection reactions in combination with FK506.

## Abbreviations

Sirt1: sirtuin1
CNIs: calcineurin inhibitors
APC: antigen-presenting cell
HDAC: histone deacetylase
NAD: nicotinamide adenine dinucleotide
DMSO: dimethyl sulfoxide
ELISA: enzyme-linked immunosorbent assay
MHC: major histocompatibility complex
GVHD: graft-versus-host disease
FK506: tacrolimus
